# Editorial on Nutrition Education, Food Literacy, and Healthy Diets in Childhood and Adolescence

**DOI:** 10.3390/nu16234108

**Published:** 2024-11-28

**Authors:** Maha Hoteit, Hassan Younes, Reema Tayyem

**Affiliations:** 1 Food Sciences Unit, National Council for Scientific Research of Lebanon (CNRS-L), Beirut P.O. Box 11-8281, Lebanon; 2 PHENOL Research Program, Faculty of Public Health, Section 1, Lebanese University, Beirut P.O. Box 6573, Lebanon; 3 Institut Polytechnique UniLaSalle, Université d’Artois, IDEALISS, URL 7519, 60026 Beauvais, France; hassan.younes@unilasalle.fr; 4 Department of Human Nutrition, College of Health Science, Qatar University, Doha P.O. Box 2713, Qatar; reema.tayyem@qu.edu.qa

## 1. Introduction

Childhood and adolescence are critical developmental stages, where adequate nutrition plays a pivotal role in ensuring proper growth and long-term health [1]. Poor nutrition throughout these formative years can impair physical growth and elevate the risk of diet-related noncommunicable diseases (NCDs) later in life [2]. This underscores the pressing need for comprehensive nutrition education and food literacy programs targeted at school-aged children and teenagers.

## 2. The Significance of Food Literacy Education

School food literacy education offers a valuable opportunity within the formal education system to improve the food knowledge and skills of young people [3]. By fostering a strong understanding of nutrition, food preparation, and healthy eating habits, we empower children to achieve their maximum developmental potential. Food literacy is not just about raising awareness; it provides students with practical skills and knowledge essential for navigating various food environments [3]. This includes understanding food labels, recognizing the nutritional value of different foods, and learning how to prepare healthy meals. Such competencies enable children to make informed dietary choices, promoting healthier eating patterns that can prevent diet-related issues both in childhood and later in life.

## 3. Research and Intervention Needs

To effectively address the issue of inadequate food literacy, further research is essential. Our research team has already published several papers highlighting findings in the Arab countries [4,5]. Evaluating food literacy status, especially in regions where malnutrition and food insecurity coexist, is critical. Understanding these dynamics will inform the design of targeted interventions that cater to the specific needs of various demographics [6]. Moreover, assessing the efficacy and applicability of food literacy interventions, both in school and community settings, is crucial [7].

This editorial presents a comprehensive overview of 12 insightful articles published across five esteemed journals, collectively addressing the critical themes of nutrition education, food literacy, and healthy diets in childhood and adolescence. These articles explore various aspects of how food literacy influences dietary choices, health outcomes, and the overall well-being of young individuals. In the following sections, we will provide a summary of each article, highlighting key findings and implications for future research and practice. By synthesizing this diverse body of research, we aim to emphasize the urgent need for effective nutrition education interventions and advocate for policies that support food literacy as a fundamental component of childhood development. The first article by Kranjčec et al. explores changes in nutritional status among children and adolescents undergoing treatment for acute lymphoblastic leukemia (ALL). It reveals a connection between nutrition literacy and malnutrition, which is prevalent in this population and affects therapy outcomes. Notably, the study identifies a significant increase in obesity rates during treatment, highlighting the necessity for dietary consultations and nutritional support [8]. Martins et al. investigate the relationship between the time parents spend preparing dinner and the consumption of made-from-scratch meals by their children. Their findings indicate that more time spent on meal preparation correlates with healthier eating patterns. This suggests that family dynamics and parental involvement in cooking play a crucial role in promoting healthier diets, aligning with Kranjčec et al.’s emphasis on the importance of dietary support and food literacy [9]. In a complementary vein, Sanlier et al. focus on the influence of sociodemographic factors on nutritional knowledge and literacy among Turkish adults [10]. They find that higher levels of nutrition education correlate with improved health literacy, indicating that education can empower individuals to make healthier choices. This reinforces the findings of Martins et al., suggesting that increased cooking knowledge can lead to better dietary habits in families [9,10]. Ji and Ko’s study on the training adequacy of university food canteen employees in China highlights another dimension of food literacy [11]. Their results point to gaps in professional training, which can affect the quality of the food provided. This is crucial when considering the broader context of nutritional education, as the quality of food services makes dietary choices available to students, particularly when compared to the family-focused approaches highlighted by Martins et al. [9,11]. Chen et al. analyze the digital food environment in China, revealing that unhealthy food promotions dominate online platforms. This finding complements the insights from Kranjčec et al. and Martins et al., as it emphasizes the external factors influencing dietary choices. The pervasive promotion of unhealthy foods can undermine parental efforts to provide healthier meals, indicating a need for strategic public health interventions [8,9,12]. Song et al. explores the impact of professional feeding guidance on infants’ self-feeding during the introduction of complementary foods in Beijing [13]. The significant increase in self-feeding among infants who received guidance reflects the importance of informed feeding practices, paralleling Sanlier et al.’s findings that nutrition education positively influences dietary behaviors [10,13]. Mahajan et al. investigate sugar intake among preschool-aged children in Canada, linking sociodemographic factors to dietary choices [14]. Their findings align with the broader themes of the articles, suggesting that targeted public health interventions are necessary to address disparities in dietary habits, particularly among vulnerable populations [14]. Carrasco-Luna et al. delve into genetic factors related to obesity in a Spanish pediatric cohort, identifying specific SNPs associated with obesity-related biomarkers. This genetic perspective adds depth to the discussion, highlighting that while education and food literacy are critical, biological factors also play a role in dietary outcomes [15]. Triatmaja et al.’s study presents a systematic review on the effectiveness of the positive deviance approach to reducing malnutrition in children under five. This aligns with the practical applications discussed by Kranjčec et al. and Song et al., illustrating the importance of community-driven interventions in addressing malnutrition [8,13,16]. Salatto et al. discuss the rising incidence of new eating disorders, particularly in the post-pandemic context. This raises concerns about the psychological aspects of eating behaviors, suggesting that education and support must also address mental health, complementing the focus on nutrition literacy and healthy diets [17]. Congiu et al.’s study provides an update on complementary feeding practices in Italy, noting a shift towards baby-led weaning and traditional spoon-feeding. This evolution reflects changing parental attitudes and the need for continuous education among healthcare professionals, aligning with the broader themes of nutrition education and food literacy [18]. Finally, Dewi et al. analyze diet quality among adolescents in a post-disaster area in Indonesia, emphasizing the influence of various factors on dietary choices. This highlights the importance of tailored interventions that consider specific contextual challenges, linking back to the diverse influences on nutrition discussed across the other studies [19].

Together, these articles illustrate the multifaceted nature of nutrition education, food literacy, and healthy diets. By comparing and linking these studies, we can see how family dynamics, education, external food environments, and individual factors intertwine to shape dietary behaviors. This comprehensive understanding is essential for developing effective strategies to promote healthier eating habits among children and adolescents, ultimately leading to better health outcomes. Investing in nutrition education and food literacy is not merely an option; it is imperative for fostering healthier generations. By prioritizing research, evaluating interventions, and advocating for policy changes, we can cultivate an environment where children and adolescents thrive nutritionally. Together, let us champion the cause of food literacy and work towards a future where all young individuals have the knowledge and skills to lead healthy lives.
